# Nano-Sized and Filterable Bacteria and Archaea: Biodiversity and Function

**DOI:** 10.3389/fmicb.2018.01971

**Published:** 2018-08-21

**Authors:** Lydia-Ann J. Ghuneim, David L. Jones, Peter N. Golyshin, Olga V. Golyshina

**Affiliations:** ^1^School of Environment, Natural Resources and Geography, Bangor University, Bangor, United Kingdom; ^2^School of Biological Sciences, Bangor University, Bangor, United Kingdom

**Keywords:** nano-sized microorganisms, ultramicrocells, filterable microorganisms, unculturable, oligotrophy, copiotrophy

## Abstract

Nano-sized and filterable microorganisms are thought to represent the smallest living organisms on earth and are characterized by their small size (50–400 nm) and their ability to physically pass through <0.45 μm pore size filters. They appear to be ubiquitous in the biosphere and are present at high abundance across a diverse range of habitats including oceans, rivers, soils, and subterranean bedrock. Small-sized organisms are detected by culture-independent and culture-dependent approaches, with most remaining uncultured and uncharacterized at both metabolic and taxonomic levels. Consequently, their significance in ecological roles remain largely unknown. Successful isolation, however, has been achieved for some species (e.g., *Nanoarchaeum equitans* and “*Candidatus* Pelagibacter ubique”). In many instances, small-sized organisms exhibit a significant genome reduction and loss of essential metabolic pathways required for a free-living lifestyle, making their survival reliant on other microbial community members. In these cases, the nano-sized prokaryotes can only be co-cultured with their ‘hosts.’ This paper analyses the recent data on small-sized microorganisms in the context of their taxonomic diversity and potential functions in the environment.

## Introduction

Recent technological advances in microbiology have helped to reveal the enormous diversity of prokaryotic life on our planet (Kuczynski et al., 2010; Caporaso et al., 2011; Thompson et al., 2017). While this has enabled us to characterize and map prokaryote populations across a diverse array of ecosystems, the functional role of most of these organisms remains unknown, due to our inability to culture, and study them in the laboratory. Nevertheless, using culture-independent approaches, e.g., metagenomics, many new candidate taxa that include nano-sized and filterable organisms have been discovered.

Nano-sized microorganisms are termed ‘ultra-micro bacteria,’ ‘ultra-micro cells,’ ‘dwarf cells,’ ‘ultra-small bacteria,’ ‘nanoorganisms,’ ‘nanobacteria,’ nanoarchaea and ‘nanobes’ (Velimirov, 2001; Baker et al., 2010; Duda et al., 2012). The term nanoarchaea only relates to the phylum *Nanoarchaeota* (Huber et al., 2002), although it is commonly erroneously used within the literature. The exact definition of these terms is widely debated and no clear set of guidelines currently exists, however, it is considered that the microorganism must be in the “nano-range” (i.e., 50–400 nm) in size. It should also be noted that in regards to aquatic systems, these ultra-small-sized organisms are not part of nanoplankton (2.0–20 μm in size), but instead reside in the picoplankton (0.2–2.0 μm) or femtoplankton (0.02–0.2 μm) communities (Sieburth et al., 1978; Fenchel, 1982; Azam et al., 1983).

Previous studies have focused on detection of ultra-small-sized organisms in a wide range of environmental conditions including: acid mine drainage settings (AMD) (Baker and Banfield, 2003; Baker et al., 2006), glacial ice (Miteva and Brenchley, 2005), permafrost (Suzina et al., 2015), freshwater (Fedotova et al., 2012; Ma et al., 2016; Nakai et al., 2016), subterranean bedrock (Wu et al., 2015), hypersaline lakes (Narasingarao et al., 2012), the open ocean (Venter et al., 2004; Giovannoni et al., 2005; Glaubitz et al., 2013; Rogge et al., 2017), and the human body (Kajander and Ciftcioglu, 1998; Kajander et al., 2003; He et al., 2015). The predictions from genomic data from these environments suggest that there are many microorganisms that contain small genomes and either are present as free-living organisms or form a symbiotic relationship with other life forms, which adds another level of complexity to assess their functional role in the environment.

As the review of Duda et al. (2012) discusses a number of issues related with ultramicrobacteria, the aim of present review was to highlight the latest discoveries related to (1) taxonomic diversity, (2) biogeography, (3) current experimental approaches to characterize these organisms and (iv) potential role of ultra-small Bacteria and Archaea within a contrasting range of environments.

### Overview of Terminology

**FIGURE 1:**
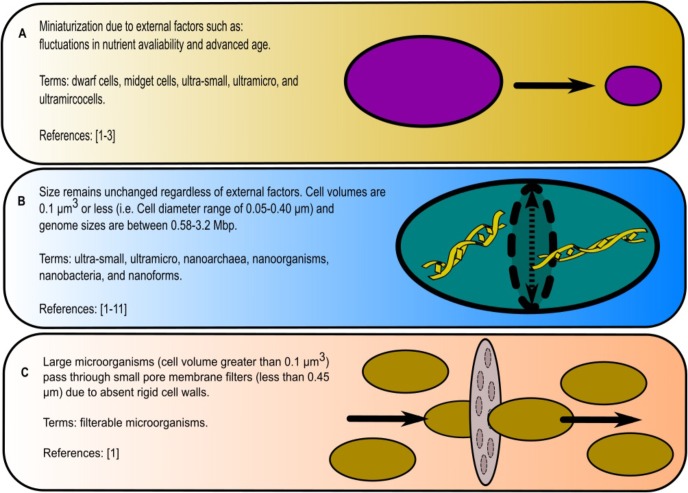
Summary of definitions used to describe nano-sized organisms: **(A)** microorganisms shrinking in body size, **(B)** consistently small-bodied microorganisms and **(C)** large microorganisms that pass through filters. References are the following: [1] Duda et al., 2012; [2] Velimirov, 2001; [3] Panikov, 2005; [4] Schut et al., 1995; [5] Miteva and Brenchley, 2005; [6] Luef et al., 2015; [7] Huber et al., 2002; [8] Rogge et al., 2017; [9] Giovannoni, 2017; [10] Kajander and Ciftcioglu, 1998; [11] Fedotova et al., 2012.

When considering ultra-small or nano-sized organisms, it is important to note the significance of the terminology. There is no singular definition of what a nano-sized organism is (ultra-small bacteria, ultra-micro bacteria, nanobes, nanoforms, ultramicrocells, etc.) and consequently a variety of interpretations exists. Many of the terms are either synonymous, as in the case of ultra-small and ultra-micro (Velimirov, 2001), or can be classified as separate organisms, as in the case of nanobacterium and nanobe (Duda et al., 2012). Here, we consider three scenarios for their denotation (**Figure 1**).

The first scenario that these microorganisms originated from known species, whose cell size decreases over time due to either internal and/or external factors such as lack of nutrients or aging (Velimirov, 2001; Panikov, 2005; Duda et al., 2012). Such ability of bacteria and archaea to change size in response to external stress is a well-studied phenomenon. For example, under low nutrient conditions, *Staphylococcus aureus* reduced its size by 40% (Watson et al., 1998; Chien et al., 2012), while the transfer of *Pseudomonas syringae* from laboratory culture media to plant leaves, induced the 50% reduction in cell size (Monier and Lindow, 2003). This size reduction is an attribute of dwarf cells, midget cells, ultra-small, ultramicro (Velimirov, 2001; Duda et al., 2012). For these cases, we advocate for the term ‘ultramicrocells’ *sensu* Duda et al. (2012).

The second scenario conjunctures that some distinct taxa, independently of growth conditions, nutrients’ availability or age of their culture do constantly exhibit small cell sizes. One source describes these organisms in the following way: the microorganisms must be 0.1 μm^3^ or smaller (<0.05–0.40 μm in diameter); the size must stay consistent under environmental stressors and life cycles; and finally, its genome size must be within the range 0.58–3.2 Mbp (Duda et al., 2012). Under this definition, nano-sized microorganisms are associated with terms like ultra-small, ultramicro, nanoarchaea, nanoforms, nanoorgansims, and nanobacteria (Schut et al., 1995; Kajander and Ciftcioglu, 1998; Velimirov, 2001; Huber et al., 2002; Miteva and Brenchley, 2005; Panikov, 2005; Comolli et al., 2009; Duda et al., 2012; Fedotova et al., 2012; Luef et al., 2015; Giovannoni, 2017; Rogge et al., 2017). However, many standard-sized microorganisms (i.e., cell volumes > 0.1 μm^3^) also possess small genomes (1.5–2.0 Mbp) and would therefore fall into the ‘ultra-small’ category if based on these criteria alone.

The third scenario are microorganisms that have the ability to pass through membrane filter pores with small diameters (0.45 or 0.22 μm) despite having larger cell sizes (above the dimensions of 50–400 nm previously mentioned) (reviewed in Duda et al., 2012). This is often due to the absence of a rigid cell wall, which allows these microorganisms to effectively squeeze through small pores and as a result are commonly confused with nano-sized or ultramicro-sized. ‘Filterable’ microorganisms is the most appropriate term to define such microorganisms.

**FIGURE 2:**
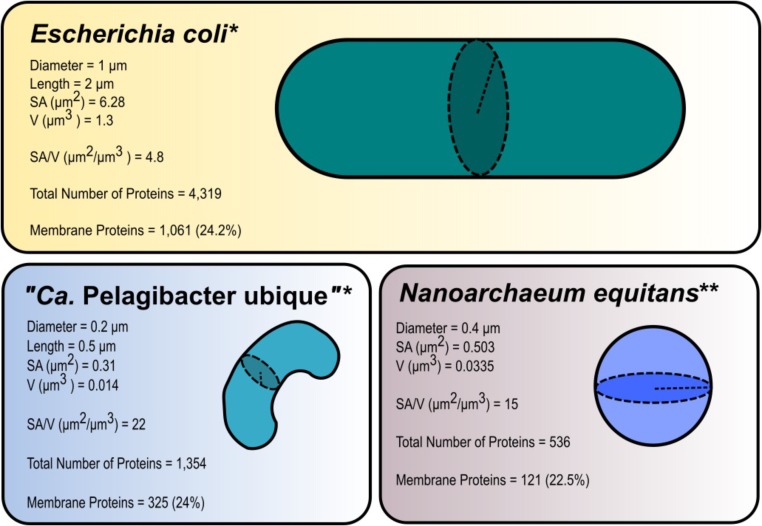
Surface area (SA) and volume (V) ratios in three selected species of different sizes: *Escherichia coli*, “*Candidatus* Pelagibacter ubique,” and *Nanoarchaeum equitans*. The microorganism with the smallest dimensions (“*Ca.* P. ubique”) had the largest ratio at 22. The habitat of “*Ca.* P. ubique” is the open ocean (oligotrophic environment) and hence its high SA/V ratio is advantageous to living in low nutrient conditions. The total protein numbers in encoded by genomes of *E. coli* (NCBI Reference Sequence: NC_000913.3), “*Ca*. P. ubique” (GenBank: CP000084.1), and *N. equitans* (GenBank: AE017199.1) are given and related with the proteins with membrane-spanning domains. For prediction of transmembrane helices in proteins, above genomes were analyzed using TMMHMM 2.0 Server at http://www.cbs.dtu.dk/services/TMHMM/ (Krogh et al., 2001; Möller et al., 2001). ^∗^Dimensions and calculations of surface area and volume were obtained from Young (2006). ^∗∗^The diameter was obtained from Huber et al. (2002), the equations for the surface area (SA = 4πr^2^, where r is the radius) and volume $V=\frac{4}{3}\pi r^{3}$, where r is the radius) of a sphere.

In this review, a unified definition for nano-sized organisms is proposed. We define them as microorganisms that exhibit constant dimensions of 50–400 nm (volume ≤ 0.1 μm^3^). All microorganisms with synonymous names that fall under the definition provided are considered nano-sized organisms. Viruses and prions, which are smaller than 50 nm in size, are not considered to be living organisms (**Figure 2** and **Table 1**). In aquatic systems, nano-sized organisms are a part of the picoplankton and femtoplankton communities, along with viruses (Sieburth et al., 1978; Venter et al., 2004; Tringe et al., 2005; Salcher, 2014).

**Table 1:** An overview of small-sized and filterable organisms denoting average cell size, average genome size, environment, separation technique (filter pore sizes), cultivability, affiliation to a confirmed species, lifestyle (free living or host-dependent), and corresponding references.

*Small-sized organism(s)*	*Environment*	*Average genome size (Mbp)*	*Average/range cell size*	*Free-living?*	*Filter(s) pore size used*	*Cultured?*	*Validly published Species*	*Reference*
‘*Ca.* Pelagibacter ubique’	Open ocean	1.3	0.01 μm^3^ (volume)	Yes	0.2 μm	Yes	Yes^∗∗∗^	Rappé et al., 2002; Giovannoni et al., 2005; Carini et al., 2012; Giovannoni, 2017; Zhao et al., 2017
*Nanoarchaeum equitans*	Submarine hot vent	0.5	0.4 μm (diameter)	No	None	Yes^∗∗^	Yes	Huber et al., 2002; Waters et al., 2003; Jahn et al., 2008
Ultrasmall microorganisms	120,000 year old Greenland ice core	NA	<0.10 μm^3^ (volume)	NA	0.4, 0.2, and 0.1 μm	Yes^∗^	No	Miteva and Brenchley, 2005
ARMAN cells	Acid mine drainage biofilm	1	0.03 μm^3^ (volume)?	Inconclusive	0.45 μm	Yes^∗∗^	No^∗∗∗^	Baker et al., 2006, 2010; Comolli et al., 2009; Comolli and Banfield, 2014; Golyshina et al., 2017
*‘Ca.* Nanobsidianus. stetteri’	Obsidian Pool, Yellowstone National Park	0.651	NA	No	0.4 μm	No	No^∗∗∗^	Podar et al., 2013; Munson-McGee et al., 2015
Oral TM7 *‘Ca.* Saccharibacteria’	Human oral cavity	0.705	200–300 nm (diameter)	No	0.22 μm	Yes	No	He et al., 2015
*‘Ca.* Nanopusillus acidilobi’	Cistern Spring, Yellowstone National Park	0.605	100–300 nm (diameter)	No	0.1 μm	Yes	No^∗∗∗^	Wurch et al., 2016
WWE3/OP11/OD1 groundwater ultra-small bacteria	Anoxic aquifer	0.878 (WWE3)	0.009 μm^3^ (volume)	No	1.2, 0.2, and 0.1 μm	No	No	Wrighton et al., 2012; Kantor et al., 2013; Luef et al., 2015
		0.694 (OD1)						
		0.820 to 1.050 (OP11)						
*‘*Nanobacterium sanguineum’	Human and bovine blood	NA	50 nm (diameter)	NA	0.1 μm	Inconclusive	No	Kajander and Ciftcioglu, 1998; Cisar et al., 2000; Kajander et al., 2003
Fossil remains	Meteorite ALH84001	NA	10–200 nm (length)	NA	NA	NA	No	McKay et al., 1996; Gibson et al., 2001
SUP05 Bacteria	Pelagic redox zones	1.164–1.53 Mbp	0.01–0.09 μm^3^ (volume)	Yes	0.2 μm	No	No	Glaubitz et al., 2013; Rogge et al., 2017; Shah et al., 2017
Filterable forms	Lake Motykino and Lake Dubrovskoe (Peatland bog)	NA	0.3–0.5 μm (rod diameter)	NA	0.22 μm	No	No	Fedotova et al., 2012
*Aurantimicrobium minutum* Str. KNCT	River water	1.62	0.04–0.05 μm^3^ (volume)	Yes	0.22 μm	Yes	Yes	Nakai et al., 2016
*Curvibacter* sp. Str. PAE-UM	River sediment	3.28	<0.05 μm^3^ (volume)	Yes	NA	Yes	Yes	Ma et al., 2016
Free-living Ultramicroscopic bacteria	Natural biotopes (i.e., permafrost, oil slime, soil, lake silt, thermal swamp moss, *Xenopus laevis*, skin)	1.5–2.4	0.02–1.3 μm^3^ (volume)	Yes	NA	No	No	Suzina et al., 2015
*Bdellovibrio spp.*^†^	NA	3.78	0.13 μm^3^ (volume)	No	NA	Yes	Yes	Duda et al., 2012
*Micavibrio admiranndus*^†^	NA	NA	0.05 μm^3^ (volume)	No	NA	Yes	Yes	Duda et al., 2012
*Vampirovibrio chlorellavorus*^†^	Reservoir water	NA	0.3–0.6 μm (diameter)	No	NA	Yes	Yes	Duda et al., 2012
*Kaistia adipata, str. NF1, NF3*^†^	Soil and lake sediment	2.4	0.1–0.5 μm^3^ (volume)	No	0.22 μm	Yes	Yes	Duda et al., 2012
*Chryseobacterium solincola, str. NF4, NF5*^†^	Soil and lake sediment	1.7	<0.1 μm^3^	No	0.22 μm	Yes	Yes	Duda et al., 2012


*NA denotes information not available within the respective source. Some studies showed that the results were inconclusive, meaning that there were conflicting conclusions in the literature. ^∗^Colonies were slow-growing taking up to a few months to become visible. ^∗∗^Can be co-cultured in association with the host. ^∗∗∗^Proposed Candidatus status. ^†^Parasitic ultramicrobacteria discussed in Duda et al. (2012) review.*

## Microbial Adaptations

In the natural environments microorganisms use an arsenal of mechanisms to cope with, and adapt to, constantly changing physio-chemical conditions, through changes in their gene expression profile, physiology and morphology (Schut and Jørgensen, 2001; Chien et al., 2012). Here we highlight various survival strategies in prokaryotes, knowledge of which may stimulate future discoveries pertaining to small-sized organisms.

### Extreme Small Size

In general, microorganisms do not fit into one standard model of size or shape (morphology) due to the impact environmental stressors (Young, 2006; Chien et al., 2012; Lever et al., 2015; Cesar and Huang, 2017). The efficiency of nutrients’ uptake is dependent on organism size and the number of transporter systems on its surface (Button et al., 1998). Hence, in the case of cell size reduction, the surface area-to-volume ratio tends to increase (**Figure 2**). This, however, does not imply that the percentage of genes encoding membrane-bound proteins in genomes is higher in organisms with a larger surface area-to-volume ratio (Stevens and Arkin, 2000) (**Figure 2**).

Under conditions of starvation and energy limitations, microorganisms can drastically decrease in size, alter cellular morphology and motility to increase survivability (Torrella and Morita, 1981; Lever et al., 2015; Cesar and Huang, 2017). For example, in low organic phosphate conditions, *Caulobacter* spp. increase their surface area to volume ratio by growing a prosthecae, stalk-like protrusions, in order to enhance organic phosphate uptake (Wagner et al., 2006; Lever et al., 2015). Another example is the species *Sphingomonas alaskensis*, which also undergoes morphological changes in response to the fluctuations in nutrients availability. In natural pelagic environment its body size is quite small (diameter: 0.2–0.5 μm; length: 0.5–3 μm) yet when grown on nutrient rich trypticase soy agar medium it increases in both diameter and length (diameter: 0.8; length: 2–3 μm) (Vancanneyt et al., 2001; Lever et al., 2015).

### Lifestyle: Free-Living vs. Symbionts

Nano-sized organisms are thought to contain genomes coding for a very limited number of functions and pathways, which is a characteristic commonly associated with symbionts, however, nano-sized organisms do also exist in a free-living state. Generally, symbionts do not have the means for their existence without relying on essential metabolites provided by the host. However, these organisms do thrive probably due to their highly specialized and unique functions which allows the host to be more competitive (McCutcheon and Moran, 2011). For instance, TM7 (“*Ca*. Saccharibacteria”) bacteria isolated from the human oral mucosa can effectively conceal its host, *Actinomyces odontolyticus* subsp. *actinosynbacter* XH001, from the human immune system response (He et al., 2015; further discussion in Section “TM7 Bacteria or “*Candidiatus* Saccharibacteria””).

### Oligotrophy and Copiotrophy

Oligotrophs also known as K-strategists, are organisms that prefer low-nutrient environments (Schut et al., 1997; Panikov, 2005; Torsvik and Øvreås, 2008). One of the most well-characterized oligotrophic environments is the open ocean, which encompasses 90% of the biosphere (i.e., the sum of all the ecosystems) (Schut et al., 1997; Hansell et al., 2009). In this environment, many essential nutrients are only present in very low concentrations: iron at 0.2–1.38 nmol kg^-1^, nitrate at 1.04 μmol kg^-1^, phosphate at 0.074 μmol kg^-1^, silicate at 3.2 μmol kg^-1^, dissolved inorganic carbon at 11 μmol kg^-1^, and dissolved organic carbon (DOM) at 40–80 μmol kg^-1^ (Johnson et al., 1997; Roshan and DeVries, 2017; Sauzède et al., 2017; Tagliabue et al., 2017), which makes it difficult to mimic such conditions and obtain a detectable growth of these microorganisms *in vitro*. At such low concentrations of nutrients microorganisms lower their metabolic rates and become less capable of forming aggregates (i.e., colonies), as seen in many pelagic organisms, such as SUP05 group bacteria and in “*Ca*. Pelagibacter ubique” (see references below in Sections “SUP05 Group” and “*Ca*. Pelagibacter ubique”). Overall, oligotrophs are characterized by small cell sizes, which are more advantageous in low nutrients conditions. The correlation between oligotrophy and diminutive size appears almost ubiquitously (Giovannoni et al., 2014), however, few studies have detected ultra-small-sized microorganisms in high-nutrient systems, such as eutrophic aquifers or the human oral cavity (He et al., 2015; Luef et al., 2015).

Copiotrophs or R-strategists, are active, fast-growing with larger cell body sizes, usually motile organisms well-suited to nutrient-rich environments; they represent the majority of bacteria and archaea cultured up to date (Dang and Lovell, 2016; Giovannoni, 2017). Despite being easy to culture, copitotrophs appear as rarer taxa in natural environments. They take advantage of sporadic high nutrients concentrations which in turn may transiently cause a rapid population growth (Vergin et al., 2013; Dang and Lovell, 2016). It is thought that copiotrophs are not nano-sized organisms as an increased surface area-to-volume ratio is not necessarily advantageous in nutrient-rich environments (Martínez-Cano et al., 2015). However, copiotrophic bacteria also tend to reduce their sizes as a response to starvation conditions in an attempt to increase their surface area-to-volume ratio, as in the case of *S. aureus* (40% reduction in size) and *P. syringae* (50% reduction in size) (Watson et al., 1998; Monier and Lindow, 2003).

## Characterization

Due to the constraints in accurately mimicking environmental settings *in vitro*, the cultivation of small organisms is often problematic and represents a main bottleneck in the process of their phenotypic characterization. In order to predict functional traits of nano-sized microorganisms as a part of the microbial community, culture-independent techniques are currently employed as primary approaches, as stand-alone or combinations of approaches: metagenome sequencing, flow-cytometry and fluorescence microscopy. Below is a brief overview of some culture-independent techniques and the challenges that arise when attempting to isolate nano-sized microorganisms.

### Metagenomics

As indicated above, metagenomics has played a central role in attempts to detect small-sized and filterable organisms and elucidate their functions. In turn, the isolation and characterization of nano-sized organisms has yielded, and to some extent, validated new genomic data (Huber et al., 2002; Giovannoni et al., 2005). In many of the large-scale metagenomics studies, the significant proportion of assembled genomes exhibited small sizes (Rappé et al., 2002; Venter et al., 2004). In particular, an in-depth investigation of the SAR11 clade led to the discovery of “*Ca*. Pelagibacter ubique,” a ubiquitous and predominant marine bacterium (Giovannoni, 2017; Zhao et al., 2017). Also, microbial communities in the deep biosphere proved to be more diverse than previously anticipated, with a plethora of miniature cells with small genomes (Wu et al., 2015). Finally, hypersaline lakes, a good model for extreme habitats, were found to contain filterable cells, about 0.6 μm in diameter, that were termed “*Ca.* Nanohaloarchaeota” (Narasingarao et al., 2012). This study was in large facilitated by a more targeted sample preparation (filtration) procedure and *de novo* sequencing approach. However, we must note that small genomes and the ability to pass via 0.1, 0.22, and 0.45 μm pore-size filters are not necessarily the evidence of small sizes of microorganisms (i.e., filterable microorganisms), for instance, the symbiont “*Ca.* Tremblya princeps” has an extremely reduced genome of 0.13 Mbp, yet, examination by microscopy showed its length to be ca. 2.3 μm (McCutcheon and Moran, 2011).

### Flow Cytometry and FACS Cell Sorting

The further culture-independent techniques, flow cytometry (Gasol and Morán, 1999; Miteva and Brenchley, 2005; Wang et al., 2007; Neuenschwander et al., 2015) and fluorescence *in situ* hybridization (FISH) (Glaubitz et al., 2013; Munson-McGee et al., 2015; Neuenschwander et al., 2015) have been widely used to study microbial populations in their natural environments. In combination with fluorescence probes targeting SSU rRNA or immunolabeling cellular proteins, this approach allows quantification of a certain taxonomic group of microorganisms (Neuenschwander et al., 2015). Combining FISH/CARD-FISH (Fluorescence *in situ* Hybridization/Catalyzed Reporter Deposition-Fluorescence *in situ* Hybridization) and flow cytometry (also known as 2C-FISH) allowed for sorting and obtaining relatively pure populations of microorganisms, as it was the case of LD12 clade of ultramicrobacteria from freshwater. These ultramicrobacteria were known to be very difficult to isolate and characterize due to their small genomes and hence limited metabolic repertoires, cell sorting was therefore the crucial starting point for their subsequent genomic studies (Salcher et al., 2013; Neuenschwander et al., 2015). Although improvements in individual techniques were achieved in this study, the methodology of sample preparation is still tedious and time-consuming with relatively limited yields of cells (Neuenschwander et al., 2015). Whatever the case, the applications of cell sorting have been successful in resolving a number of “single-cell-genomes” (Ishoey et al., 2008; Probst et al., 2018).

### Isolation of Nano-Sized Microorganisms

Although isolation is an essential step in characterizing organisms, it is often overlooked and traditional approaches to culture them frequently prove unsuccessful. Many of the studies presented in this review employed filtering through 0.1–1.2 μm pore size filters to facilitate enrichment and isolation (**Table 1**). The exception to the filtration methodology was *Nanoarchaeum equitans*, which was co-cultured with the host, *Ignicoccus hospitalis*, and then separated out via centrifugation (Huber et al., 2002; Waters et al., 2003). Conversely, while the target microorganisms may be small enough to pass through the membrane, certain larger organisms can squeeze through pores, due to a lack of rigidity of their cells. Another example of organisms squeezing through small-sized pores are archaea of families *Ferroplasmaceae* (0.2–3 μm in diameter in average) and *Thermoplasmataceae* (0.5–3 μm in length and 0.2–0.5 μm thick), that can easily pass through a <0.45 μm pore filter due to the lack of a rigid cellular envelope (Golyshina, 2014; Nagy et al., 2016).

In previous studies, along with ‘small-sized-organisms,’ many other microorganisms have been co-isolated (Venter et al., 2004; Tringe et al., 2005; Garza and Dutilh, 2015). An extra level of authentication is therefore necessary to reliably confirm the existence and metabolic function of these organisms, e.g., through an improvement in isolation and culturing techniques. Small cell size is the only certainty related to nano-sized organisms that belong to a range of taxa and do not share a common metabolism. For their characterization, a prior genomic analysis of the source community is critical. This would allow the targeting, e.g., organism-specific surface proteins to enable FACS- or immunoprecipitation-based techniques targeted organisms of interest.

## Nano-Sized and Filterable Microorganisms

**FIGURE 3:**
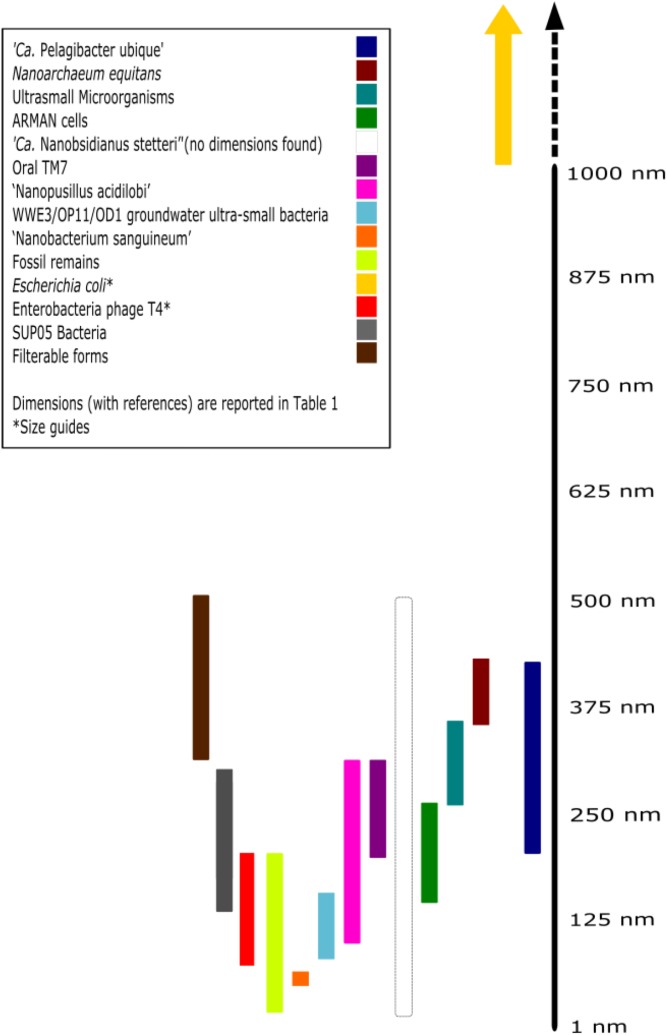
Size comparison of nano-sized organisms. Each of the colored lines represents relative range of sizes (in one dimension) of each individual. References and numerical ranges for individuals can be found in **Table 1**. If size was reported with volume, the organism was assumed to be spherical and then obtained the radius with the equation, $V=\frac{4}{3}\pi r^{3}$, where r is the radius. ^∗^References for size guides: *Escherichia coli* (approximately 1 μm × 2 μm) and phage T4 (approximately 90 nm × 200 nm) (Leiman et al., 2003). ‘*Ca.* Nanobsidianus stetteri’ has no available information concerning cellular dimensions.

Though the different characterization techniques as mentioned above, the story of ultra-small microorganisms and our understanding of their ecosystem functioning is rapidly evolving. Here, some of the major milestones are outlined in regards to successful isolation and characterization of a variety of nano-sized organisms. Further, we have summarized the data on various microorganisms covered in this section in **Figure 3** and **Table 1**.

### Rise of the Very Small

Although ultramicrobacteria have been known for a long time (Oppenheimer, 1952), the subject laid dormant for a number of years. This was in part due to the limitations in microbiological techniques, and the lack of knowledge of their physiology and metabolism. That changed when McKay et al. (1996) first claimed their existence in Martian rocks. Not only did this imply that life may exist on exoplanets, but it also challenged the ideas on lower limit of size of a lifeform (McKay et al., 1996; Gibson et al., 2001). It was suggested that the smallest free living organism must be in the spherical diameter range of 250–300 nm to properly contain the 250–300 proteins essential to life (including the ribosomal proteins), although it was also suggested that, theoretically, a primitive organism can be as small as 50 nm (Kajander and Ciftcioglu, 1998). This was similar to an earlier study by Mushegian and Koonin (1996) who hypothesized that the minimal number of genes required for life ranges between ca. 250–450, however, there was no consensus on the number of ribosomal proteins that were actually needed. Importantly, it was never established in the McKay et al. (1996) study whether these nano-scale objects were free-living organisms, nor was it confirmed that these objects were living at all.

### Nanoarchaeum equitans

Huber et al. (2002) found that a new archaeal species, *I. hospitalis*, isolated from hot submarine vents, had in its culture a companion of a small cell size. The new phylum *Nanoarchaeota* and corresponding species *N. equitans* were described as the first nano-sized archaea. The genome analysis revealed that it contained a chromosome of only 0.5 Mbp (Huber et al., 2002), while electron and fluorescence microscopy suggested that the cells of *N. equitans* were ca. 400 nm in diameter and were attached to the cell surface of its host, *I. hospitalis.* Further, it was shown that *N. equitans* was incapable of growing without its host, which in contrary neither benefited or was impaired by *N. equitans* (Huber et al., 2002; Jahn et al., 2008). The inability of *N. equitans* to survive without its host is reflected in its small streamlined genome, which was a result of massive gene losses (Huber et al., 2002) including those for key biosynthetic pathways for vitamins, cofactors and amino acids (Torrella and Morita, 1981; Mushegian and Koonin, 1996; McCutcheon and Moran, 2011).

### “ARMAN” Cells

“ARMAN” (Archaeal Richmond Mine Acidophilic Nanoorganism) were first detected through *de novo* shotgun sequencing of aqueous sample obtained from an acid mine drainage (AMD) system and not through standard PCR-based surveys (Baker et al., 2006). Subsequent cryo-TEM analysis revealed an accumulation of filterable cells that were 0.03 μm^3^ in volume with clearly defined cell walls (Comolli et al., 2009). “ARMAN” cells were initially considered free-living, possibly slow-growing, organisms possessing some intracellular tubular structures (Comolli et al., 2009), however, later on, their ability to free-living lifestyle was questioned (Comolli and Banfield, 2014).

According to the metagenome analysis with almost fully assembled “ARMAN” genomes of ca. 1 Mbp in size and proteomics, these organisms contain a rather unique set of genes with 45% of the genes failing to match to a known biological function, while 63% of the proteins identified could not be assigned to known archaeal protein families (Baker et al., 2010). Due to the small sizes of their genomes, it was assumed that “ARMAN” cells are certainly dependent on other community members, being either symbionts or commensals (Baker et al., 2010).

Cultivation of an “ARMAN”-related organism, ‘*Ca.* Mancarchaeum acidiphilum’ Mia14 revealed that it was dependent on its host, euryarchaeon *Cuniculiplasma divulgatum* (Golyshina et al., 2017). As in the above examples, Mia14 underwent streamlining of its genome (0.95 Mbp) due to the massive gene loss. Similarly, it exhibits significant voids in its biosynthesis of amino acids, CoA, NAD and NADP, vitamins and heme. Additionally, its central metabolism lacks glycolysis and gluconeogenesis, pentose phosphate pathway and tricarboxylic acid cycle (Golyshina et al., 2017). Interestingly, Mia14 cell sizes were only marginally smaller than *Cuniculiplasma* cells, which were 0.1–2 μm in size (Golyshina et al., 2016).

### Other *Archaea*

“*Candidatus* Nanobsidianus stetteri” Nst1, a member of phylum *Nanoarchaeota* was first reported after the single-cell isolation alongside its host from the order *Sulfolobales* (phylum *Crenarchaeota*) by Podar et al. (2013). Unlike *N. equitans*, which is associated with a single host species, *I. hospitalis*, “*Ca.* N. stetteri” can use a multitude of *Sulfolobales* species as hosts. Its genome was ca. 20% larger than that of *N. equitans* and possessed a complete gluconeogenesis pathway (Podar et al., 2013; Munson-McGee et al., 2015). The genome analysis also indicated that “*Ca*. N. stetteri” genome coded for cellular functions previously not associated with the *Nanoarchaeota* taxon; the study concluded that these archaea share a common ancestor with *N. equitans* (Podar et al., 2013; Munson-McGee et al., 2015). Another study (Munson-McGee et al., 2015) has partially resolved two further single-cell genomes of “Nanobsidianus”-related archaea from Yellowstone hot springs and suggested their close relatedness with “*Ca.* N. stetteri” Nst1, but pointed at their association with archaea of “Acidicryptum spp.” of *Sulfolobales*. “*Ca.* Nanopusillus acidilobi” is another success story, where this small-sized, reduced-genome archaeon was co-cultured with its host, *Acidilobus* sp. A7 by Wurch et al. (2016). “*Ca.* Nanopusillus acidilobi” is a thermophilic ectosymbiont, much like *N. equitans* and “*Ca*. Nanobsidianus stetteri.” This particular species is only marginally smaller in body size than *N. equitans* (approximately 100–300 nm in diameter), both share approximately 80% SSU rRNA gene sequence identity (and 97–98% with ‘*Ca*. Nanobsidianus stetteri’), and exhibit much of the same functions as judged from genomic data (Wurch et al., 2016). “*Ca.* Nanopusillus acidilobi” genome possesses no genes related to respiration, ATP synthesis and cannot produce its own amino acids, lipids, nucleic acids, and co-factors. Genomic data suggests that, like in its relative, “*Ca.* N. stetteri,” glycogen may serve as a storage compound and facilitate its short-term energetic independence from the host (Wurch et al., 2016). A high density of “*Ca.* Nanopusillus acidilobi” on the surface of its host *Acidilobus* sp. 7A, deficiency of its genome in genes for central metabolic, biosynthetic and energy-generating pathways suggest a commensal or ectoparasitic lifestyle of these nanoarchaea (Wurch et al., 2016). Expression of flagellar proteins reported in proteomic data further suggests that “*Ca.* Nanopusillus acidilobi” has the ability to migrate from one host to another (Wurch et al., 2016).

### “*Ca.* Pelagibacter ubique”

While the existence of oceanic ultramicrobacteria has been well-documented, obtaining them in a pure culture remained difficult. Earlier studies (Morris et al., 2002; Rappé et al., 2002) revealed a very abundant clade of *Alphaproteobacteria*, SAR11, which makes up to 25% of plankton in the open ocean and is represented by small-sized, simple-metabolism bacteria (Giovannoni, 2017). Initially found in pelagic water sampled from the Sargasso sea, these bacteria termed “*Ca*. Pelagibacter ubique” had genomes of approximately 1.3 Mbp and are considered to be one of the smallest free living cells (Giovannoni, 2017; Zhao et al., 2017). Their genomes contained the necessary gene sets for producing all 20 amino acids as well as other essential biosynthetic pathways (Giovannoni et al., 2005; Carini et al., 2012). Subsequent studies indicated that “*Ca*. P. ubique” required an unconventional medium, which was composed of methionine, glycine, pyruvate, and artificial seawater (Carini et al., 2012).

It was also found that “*Ca*. P. ubique” had a rather unique metabolism because of its ability to use glycolate instead of glycine at low glycine concentrations. Glycolate can be used in glycine biosynthesis through glyoxylate amination, with the glycine consequently being used for serine biosynthesis (Carini et al., 2012; Tripp, 2013). The glycolate to serine pathways are regulated by two glycine riboswitches, the first of which controlling the glyoxylate to glycine biosynthesis and the second regulating the glycine to serine biosynthesis. At low glycine concentrations, the first riboswitch is turned on to produce more glycine (Tripp, 2013). When there are ample amounts of glycine in the cell, the first riboswitch turns off the glycine biosynthesis and the second riboswitch induces the conversion of glycine to serine. The ability to use glycolate instead of glycine to further create serine may be an evolutionary response to relative excesses of glycolate formed by phytoplankton in carbon limited conditions (Carini et al., 2012). As a free-living organism, “*Ca*. P. ubique” has the ability to adapt to changing conditions fairly well-despite having a streamlined genome. It also challenged the previous assumption that small genome sizes were restricted to symbiotic organisms (Huber et al., 2002; Giovannoni, 2017).

### SUP05 Group

Oxygen-depleted zone in pelagic systems with dissolved oxygen concentrations below 60 μmol kg^-1^ present a unique challenge to organisms moving through the transition zone from high to low nutrient availability (Glaubitz et al., 2013; Rogge et al., 2017). According to cell counts from flow cytometry, SUP05 bacteria are a common bacterioplankton component in depleted oxygen zones (Glaubitz et al., 2013; Rogge et al., 2017). As chemolithoautotrophic organisms, they metabolize sulfur compounds and play a key role in the carbon, sulfur and nitrogen cycles to facilitate life in the redoxclines across the globe (Glaubitz et al., 2013; Rogge et al., 2017; Shah et al., 2017). They have the ability to carry out denitrification and uptake carbon dioxide in pelagic low oxygen zones, which is supported by genomic predictions, radioisotopic data and cultivation attempts (Glaubitz et al., 2013; Rogge et al., 2017; Shah et al., 2017). Cultivation attempts of one of the members of the SUP05 group, “*Candidatus* Thioglobus autotrophicus,” revealed the utilization of ammonium under anaerobic conditions and nitrite production (Shah et al., 2017). Studies on the SUP05 group have suggested cellular volumes ranging within 0.01–0.09 μm^3^ and a genome of 1.164–1.53 Mbp, which indicates that these bacteria have undergone streamlining in their evolutionary past, much like “*Ca*. P. ubique” (Rogge et al., 2017; Shah et al., 2017).

### Filterable Forms in Peatland Bogs

Despite the abundance of organic carbon in aquatic subsystems of peatland bogs, its mineralization is very slow due to the elevated concentrations of phenolic compounds causing acidification (pH 4.4–4.8), enzyme inhibition and nitrogen limitation (Fedotova et al., 2012). This is the case for sphagnum peatland bogs in northern Russia, that contain a high number of filterable bacteria and archaea, 1.69 ± 0.53 × 10^4^ and 3.16 ± 0.43 × 10^4^ cells/mL, correspondingly (Fedotova et al., 2012). Phylogenetic analysis of 16S rRNA genes shows they were derived from several phyla (Fedotova et al., 2012). One-third of the archaeal sequences had a high identity (94–99%) with representatives of the orders *Methanobacteriales* and *Methanosarcinales*, while the rest exhibited a distant relatedness (71–74% sequence identity) to cultured methanogens and collectively belonged to the LDS (Lake Dagow sediment) cluster (Glissmann et al., 2004). All detected bacterial species had high SSU rRNA gene sequence identities (94–99%) to the *Betaproteobacteria*, *Gammaproteobacteria*, *Alphaproteobacteria*, and *Actinobacteria*, which confirms that small size is an adaptation to low nutrient conditions common across the broad range of higher taxa. The study also attempted to culture filterable microorganisms on solid media: from the total microscopic cell count numbers, only a fraction of approximately 0.5–1.2% did form colonies represented by bacterial genera *Mesorhizobium, Bradyrhizobium, Sphingomonas*, and *Agrobacterium*. A major discrepancy between the SSU rRNA amplicon libraries sequences of microbial communities in those freshwater samples and the taxonomy of cultured bacteria was also observed (Fedotova et al., 2012).

### Ultra-Small Bacteria From Greenland Ice

Glacial ice presents a rather unique challenge to many microbial species due to its sub-zero temperatures and oligotrophic conditions and is considered a freshwater-like habitat for microorganisms (Hodson et al., 2008). It has been previously noted that a number of ultrasmall organisms have been detected in several ice cores (Miteva, 2008). A plethora of bacteria in 120,000 year-old Greenland ice, which, after melting the ice cores, passed through filters with pore sizes of 0.4, 0.2, and even 0.1 μm was detected (Miteva and Brenchley, 2005). Scanning electron microscopy and flow cytometry confirmed that the filtration methodology was effective at removing larger cells residing in the melted ice water. The authors also stated that a considerable amount of fungal colonies were also present, although these were not discussed in further detail (Miteva and Brenchley, 2005), however, one can assume those were derived from filterable fungal spores. It is not clear if all >1,200 cultured bacteria were ultra-small, as there was evidence of larger organisms (e.g., spores of fungi and of *Firmicutes*), which possibly were cultured due to the non-uniform sizes of filter pores, over-pressurizing filtration units or non-rigid cell envelops of microorganisms that allowed them passing through filters (Wang et al., 2007, 2008). Whatever the case, the study of Miteva and Brenchley (2005) clearly demonstrated the viability in and cultivability of very small microorganisms with experimentally measured average volumes ranging between 0.043 and 0.1 μm^3^ from, a polar ice environment.

### WWE3, OD11, and OP1 Candidate Phyla of Ultra-Small Bacteria From Groundwater

Much of the bacterial species discussed so far have been identified in oligotrophic environments, however, ultra-small organisms are not exclusive to these habitats. The WWE3-OD11-OP1 candidate phyla of groundwater bacteria were found in an eutrophic environment (Luef et al., 2015). Although these bacteria have not been cultivated, ultra-small cells have been successfully imaged challenging previous ideas on possible habitats of these organisms.

Luef et al. (2015) described the cellular structures present within ultra-small-sized-organisms: using cryo-TEM images they identified pili, cell walls, cellular division, and the presence of viruses. The study investigated the freshwater collected from an anoxic, organic carbon rich groundwater located several meters below the surface. Until that point, small-sized microorganisms were thought to be either associated with oligotrophic conditions or microbial communities with a reduced diversity, e.g., AMD. Importantly, it appears that small size can also be beneficial in other environments. The study was unable to successfully perform CARD-FISH on the proposed ultra-small cells (Luef et al., 2015) and therefore could not confirm that small cells seen were indeed of the candidate phyla that they reported on.

Metagenomic analyses by Wrighton et al. (2012) and Kantor et al. (2013) have revealed that WWE3, OP1, OD11, TM7, and SR1 candidate phyla of bacteria possessed small genomes, lacked genes for several essential metabolic processes and contained genes of both archaeal and bacterial origin. The genomic predictions inferred that WWE3, OP1, and OD11 candidate phyla are capable of growing in organic carbon-rich environments (Wrighton et al., 2012; Kantor et al., 2013; Luef et al., 2015). The RuBisCO (type II/III ribulose-1, 5-biphosphate carboxylase-oxygenase), which was predicted in these groundwater ultrasmall bacteria, is not likely to be involved into the classical CBB (Calvin-Benson-Bassham) pathway, but into the CO_2_ fixation linked with the AMP (adenosine monophosphate) recycling for ultimate ATP (adenosine triphosphate) production, similarly to the type III archaeal RuBisCo (Wrighton et al., 2012; Kantor et al., 2013). The occurrence of this pathway suggests that these organisms are not restricted to oligotrophic environments, but can survive with higher levels of available nutrients.

### TM7 Bacteria or “*Candidatus* Saccharibacteria”

Recent studies have shown that nano-sized organisms can also be a component of the human microbiome. A member of the bacterial candidate phylum TM7 (“*Ca*. Saccharibacteria”) was cultivated and co-isolated with *A. odontolyticus* subsp. *actinosynbacter* strain XH001 by He et al. (2015). Having spherical cells of 200–300 nm in diameter and a genome of 0.705 Mbp, this bacterium of phylotype TM7 (strain TM7x) is associated with human oral microflora and was found to have a rather unique lifestyle. Like many of others discussed here, it is dependent on its basibiont, the host of the epibiont, an organism that resides on the surface of the host, *A. odontolyticus* subsp. *actinosynbacter* XH001. Under normal conditions, TM7x is an obligate epibiont, but during starvation it changes its lifestyle to parasitic, which eventually kills its own host and which is not usual for oral microorganisms (He et al., 2015). Additionally, TM7x lacks the ability to produce its own amino acids which further suggests its dependence on *A. odontolyticus* subsp. *actinosynbacter* XH001 (He et al., 2015). Its relationship with the host is thought to exacerbate oral mucosal diseases by concealing host immune responses by inhibiting *A. odontolyticus* XH001-induced TNF-α mRNA expression in macrophages (He et al., 2015). However, not all Candidate phylum TM7 members reside in the oral mucosa like TM7x: for example, RAAC3 with a small (0.845 Mbp) genome was originally found in a sediment obtained from an acetate-stimulated aquifer (Kantor et al., 2013). Another representative of TM7 group, “*Candidatus* Saccharimonas aalborgensis,” with the genome of 1.0 Mbp was obtained from the activated sludge bioreactor (Albertsen et al., 2013; He et al., 2015). It remains unclear why TM7x has a more streamlined genome than the other phylotypes, a possible explanation of this adaptation is its specific human microbiome habitat and its complete dependency on its actinomycete host.

## Selective Pressures for Small Size

An important conclusion that can be made from the aforementioned studies on small-size microorganisms is that their sizes and distribution are a direct consequence of nutrient availability. As mentioned previously, increasing the surface area-to-volume ratio, which is an attribute of smaller cells, provides microorganisms with the ability to take up nutrients more efficiently (Giovannoni et al., 2014). Both symbiotic and free-living organisms seem to have benefited from this change. The results from existing studies suggest that in environments with high nutrient concentrations, a nano-sized organism will likely be a symbiont (or epibiont) with a decreased cell size being a result of limited metabolic capabilities with complete metabolic dependence on a host (Martínez-Cano et al., 2015). *N. equitans* is a good example of this, as hydrothermal vents are relatively nutrient-rich, but these archaea are completely dependent on *I. hospitalis* (Giannone et al., 2014). As nutrients become less available, the more likely the small-sized organism will be free-living because an increased surface-area-to-volume ratio is incredibly advantageous under such conditions (Martínez-Cano et al., 2015). The species “*Ca*. Pelagibacter ubique” is a good illustration of this scenario. Residing in the nutrient-depleted open ocean, it needs to produce its own essential amino acids, vitamins, etc. to survive (Carini et al., 2012). This raises the question, as to why this typical adaptation (small size and limited metabolic capabilities) does also exist in relatively stable nutrient-rich habitats. One possibility is that there may be selective pressures coming from predatory species, especially in aquatic systems (Pernthaler et al., 2001; Simon et al., 2002; Pernthaler, 2017). In the study of Pernthaler et al. (2001), the presence of the protozoan, *Ochromonas* sp., resulted in an increasing population of members of *Actinobacteria* cluster Ac1. When an alternate protozoan predator, *Cyclidium glaucoma*, was introduced, no increase in population densities of Ac1 bacteria was observed (Pernthaler et al., 2001). Apparently, *Ochromonas* sp. prefers preys that are 0.8–4 μm in size, while *C. glaucoma* prefers those smaller than 0.8 μm. Since the Ac1 are smaller than 0.8 μm, the presence of only *Ochromonas* sp. allowed them to proliferate (Pernthaler et al., 2001). It was later found that some isolates of Ac1 were in fact ultramicro-sized (less than 0.1 μm^3^ volume) and this small size prevented them from predation by *Ochromonas* sp. strain DS (Hahn et al., 2003). Hence, large populations of small organisms may also be a response to, or the result of, protozoan grazing (Salcher, 2014).

Another driver of selection of particular organisms in the environment are viruses and phages. Phages are host-specific and in most cases infect highly populous and dense bacterial subpopulations, which allows for less competitive (e.g., slow-growing) cells to proliferate (Winter et al., 2010; Salcher, 2014). Lysis of infected cells releases nutrients into the environment and makes them available to other community members allowing for overall microbial population growth (Weinbauer, 2004; Salcher, 2014). Viruses, similarly to predators, act as population control by culling overpopulated microorganisms (“killing the winner”) while providing nutrients in the form of lysed cells to other species in the community (Weinbauer, 2004; Winter et al., 2010; Salcher, 2014).

## Functional Role of Small-Sized Organisms

As documented here, small-sized organisms are not characterized by any specific type of metabolism or taxonomic affiliation. Therefore, we assume that their functional role is not restricted and may highly vary depending on the environment and actual physio-chemical conditions. Aquatic systems are incredibly complex, as fluctuations between high and low nutrient availability are common. In marine systems, the addition of nutrients, e.g., in the form of nitrogen-rich fertilizers from agricultural runoffs, can greatly change the once oligotrophic environment into a copitrophic one, leading to harmful large scale phytoplanktonic blooms (Beman et al., 2005). Depending on concentrations of nutrients, populations of free-living small-celled microorganisms can either be enriched in R-strategists, or in K-strategists playing distinct roles in the community. K-strategists, e.g., SUP05 clade and “*Ca*. P. ubique,” are heavily involved with carbon and nitrogen cycling in oligotrophic areas (such as the open ocean and oxygen-depleted zones) (Giovannoni, 2017; Rogge et al., 2017). They are slow-growing and are widely dispersed, and rarely form colonies (Dang and Lovell, 2016; Giovannoni, 2017; Roshan and DeVries, 2017). R-strategists, e.g., Marine *Roseobacter* Clade (MRC) members and *Bacteroidetes*, are widely distributed and typically reside in nutrient-rich systems, e.g., in coastal systems (Dang and Lovell, 2016). These free-living organisms under favorable conditions grow quickly and may form large densely packed colonies and biofilms (Dang and Lovell, 2016). MRC bacteria can produce auxins and vitamins that are beneficial for algae (Dang and Lovell, 2016), whereas catabolically versatile *Bacteriodetes* play key roles in degrading high molecular weight DOM and biopolymers (Dang and Lovell, 2016).

In vertebrate systems, the role of these organisms appears variable. As seen in the case of TM7x, it may be beneficial or harmful to the host. *Actinomyces* strain XH001 normally elicits an immune response but TM7x modulates this response by either suppressing TNF-α gene expression in macrophages or “masking” it from macrophage detection altogether. However, under extended starvation conditions, TM7x can turn parasite, which leads to the host’s demise (He et al., 2015).

Much of the literature discussed in this review has focused on a few species, however, the concerted effect of the entire ultra-small-sized microbial community in ecosystem functioning remains unknown. As discussed earlier, filtration through <0.45 μm pore size filters, is a common method to isolate small cells from aqueous samples. Interestingly, ultrafiltration was considered a method of choice to preserve freshwater samples during their storage and prior the hydrochemistry analysis (Brailsford et al., 2017). 0.22 μm pore size filters were considered as a safe tool for sterilization and for effective removal of microorganisms. However, a recent study, which monitored the depletion of ^14^C-glucose, ^14^C-amino acid mixture, and ^33^P-orthophosphate in filtered and unfiltered freshwater samples showed significant activity and utilization of substrates by organisms capable of passing this barrier (Brailsford et al., 2017). The previous studies clearly support this claim, as a number of the species were able to pass through ultrafiltration membranes (e.g., Wang et al., 2008). The great abundance of small-sized organisms in aqueous environments may also be attributed to selective pressures of predator-prey-viral interactions (Salcher, 2014). As discussed, protists feed on bacterioplankton and select prey based on cell size (Pernthaler et al., 2001; Salcher et al., 2013; Pernthaler, 2017). Conversely, viruses select for high-density preys and promote generation of DOM from lysed cells (Salcher, 2014), which can then be utilized by nano-sized microorganisms.

Nutrient cycling by ultra-small-sized organisms is not restricted to aquatic environments. A number of studies have shown an active population of ultramicrobacteria within a wide range of soil types (Soina et al., 2012; Lysak et al., 2013; Dobrovol’skaya et al., 2015). It was previously thought that soil pores < 1 μm would be inaccessible to cells, leading to physical protection of organic carbon in soil. However, the potential of small-sized organisms to occupy this void space alongside their functional significance in soil remain unknown.

## Conclusion and Outlook

Discovery of small cells in the environment has reshaped our understanding of the microbial world and life on this planet. Using culture-independent tools first insights into the functionality of these organisms and a precise definition of the minimal sizes of living forms have been gained. Hence, it is reasonable to think that small-sized organisms may play a significant role in many environments. Many studies performed to date, however, have not considered the functionality of these organisms. Future studies should therefore shift their focus to understanding their physiology and function. As more ecosystems are explored and as techniques are improved, the possibility of finding small-sized organisms is increasing. Culture- independent analysis will remain a critical tool for modeling and predicting functionalities and abundance of these organisms, however, the functional analysis of their activities remains essential to validate genome-based predictions.

## Author Contributions

All authors conceived the review. L-AG searched the literature, synthesized the data, and wrote the manuscript. DJ, PG, and OG provided significant revisions to the manuscript including data interpretation and writing parts of the manuscript. All authors read the final manuscript.

## Conflict of Interest Statement

The authors declare that the research was conducted in the absence of any commercial or financial relationships that could be construed as a potential conflict of interest.
